# Transinger: Cross-Lingual Singing Voice Synthesis via IPA-Based Phonetic Alignment

**DOI:** 10.3390/s25133973

**Published:** 2025-06-26

**Authors:** Chen Shen, Lu Zhao, Cejin Fu, Bote Gan, Zhenlong Du

**Affiliations:** 1 College of Computer and Information Engineering (College of Artificial Intelligence), Nanjing Tech University, Nanjing 211816, China; 202221147064@njtech.edu.cn (C.S.); moonotus@njtech.edu.cn (C.F.); duzhl@njtech.edu.cn (Z.D.); 2 College of Artificial Intelligence, North China University of Science and Technology, Tangshan 063210, China; lk00100@stu.ncst.edu.cn

**Keywords:** voice synthesis, singing voice synthesis, audio signal analysis, artificial intelligence, phonetics, cross-lingual, audio processing, deep generative models

## Abstract

Although Singing Voice Synthesis (SVS) has revolutionized audio content creation, global linguistic diversity remains challenging. Current SVS research shows scant exploration of cross-lingual generalization, as fragmented, language-specific phoneme encodings (e.g., Pinyin, ARPA) hinder unified phonetic modeling. To address this challenge, we built a four-language dataset based on GTSinger’s speech data, using the International Phonetic Alphabet (IPA) for consistent phonetic representation and applying precise segmentation and calibration for improved quality. In particular, we propose a novel method of decomposing IPA phonemes into letters and diacritics, enabling the model to deeply learn the underlying rules of pronunciation and achieve better generalization. A dynamic IPA adaptation strategy further enables the application of learned phonetic representations to unseen languages. Based on VISinger2, we introduce Transinger, an innovative cross-lingual synthesis framework. Transinger achieves breakthroughs in phoneme representation learning by precisely modeling pronunciation, which effectively enables compositional generalization to unseen languages. It also integrates Conformer and RVQ techniques to optimize information extraction and generation, achieving outstanding cross-lingual synthesis performance. Objective and subjective experiments have confirmed that Transinger significantly outperforms state-of-the-art singing synthesis methods in terms of cross-lingual generalization. These results demonstrate that multilingual aligned representations can markedly enhance model learning efficacy and robustness, even for languages not seen during training. Moreover, the integration of a strategy that splits IPA phonemes into letters and diacritics allows the model to learn pronunciation more effectively, resulting in a qualitative improvement in generalization.

## 1. Introduction

Singing Voice Synthesis (SVS) [1,2,3], as a pivotal technology for generating natural and expressive singing voices from musical scores, has witnessed exponential growth in both academic research and commercial applications. Contemporary commercial systems (e.g., Vocaloid (V6.3.1) [4], Synthesizer V Studio R2 (V1.8.1)) primarily support only a few core languages including Japanese, English, and Chinese, but face significant challenges when processing multilingual hybrid compositions—especially in cross-cultural musical productions that require seamless language transitions. In practice, these systems typically support only three to four languages in a non-universal manner—each language is tailored to specific phonetic requirements, and adding a new language demands substantial investment. The development of new language support currently relies on redundant workflows: customization, large-scale singer-specific data collection, and iterative model fine-tuning. This paradigm incurs prohibitive costs that severely constrain language expansion initiatives, particularly for low-resource languages. A major obstacle contributing to this issue is the widespread lack of high-quality, diverse, and precisely annotated multilingual singing voice corpora. Constructing such datasets is not only prohibitively expensive but also often hindered by copyright constraints. The scarcity of data directly leads to under-trained models that struggle to capture the complex acoustic properties and subtle expressive nuances of singing, thereby limiting their generalization to unseen singers, styles, or languages and compromising the naturalness and expressiveness of the synthesized voice. Moreover, different languages typically adopt their own independent and incompatible phoneme representation systems (e.g., Pinyin for Chinese and ARPA for English). This fragmented phoneme encoding hinders the development of unified multilingual SVS models, making it difficult and inefficient to add support for new languages or to handle mixed-language singing scenarios effectively. It also presents significant challenges for achieving precise alignment between phonemes and acoustic features in singing, thereby affecting pronunciation accuracy and overall synthesis quality. To address this issue, our approach adopts the International Phonetic Alphabet (IPA), a standardized system of phonetic notation designed to represent the sounds of spoken language in a language-independent manner. IPA provides a unified set of symbols for phonemes across languages, enabling consistent and precise phonetic representation. This cross-linguistic consistency makes IPA particularly well-suited for multilingual singing voice synthesis, where accurate pronunciation and alignment are essential.

Empirical studies in speech processing consistently demonstrate that scaling multilingual training corpora significantly enhances cross-lingual generalization capabilities [5,6]. A notable advancement is a multilingual speech synthesis framework [7], which leverages a 115-language corpus with IPA-based universal symbol representation. This system employs a novel contrastive learning framework between phonemic sequences and acoustic features, achieving open-vocabulary matching through multilingual phoneme–speech alignment. Remarkably, it demonstrates robust zero-shot generalization across 95 linguistically diverse unseen languages.

However, such cross-lingual advancements remain remarkably underdeveloped in SVS research. While modern speech systems utilize shared phonetic representations across languages, singing voice generation faces unique challenges. Recent studies have commenced preliminary investigations in this domain. BiSinger [8] pioneers bilingual synthesis through Carnegie Mellon University Pronouncing Dictionary-based phoneme mapping, combining monolingual singing datasets with voice conversion techniques for Chinese–English vocal generation. Subsequent innovations like CrossSinger [9] and X-Singer [10] implement IPA-driven multilingual representation learning, architecturally integrating cross-lingual phoneme features through language-adaptive prosody predictors and singer-agnostic timbre decoders. These frameworks demonstrate measurable improvements in cross-singer generalization, though current performance metrics remain substantially below comparable speech synthesis benchmarks.

This study breaks through conventional multilingual speech synthesis paradigms by constructing an IPA-aligned cross-linguistic dataset based on GTSinger [11], thereby developing a zero-shot singing voice synthesis framework. The dataset spans four languages (Korean, English, Chinese, and Japanese) with a total duration of 40.9 h. Through systematic decomposition of IPA phonemes into letters and diacritics, we achieve synthesis capabilities for unseen languages and novel phoneme combinations. Building upon the VISinger2 architecture, we integrate Convolution-augmented Transformer (Conformer) [12] modules—an architecture renowned for combining self-attention with convolutions to capture both local and global dependencies—for modeling long-range phoneme–prosody relationships. This is paired with Residual Vector Quantization (RVQ), a multi-stage technique that discretizes continuous features into compact, structured codes, for language-agnostic codebook optimization. Specifically, RVQ encodes cross-lingual acoustic features into hierarchically composable discrete symbolic representations via multi-stage residual vector space decomposition. Compared to traditional single-stage quantization methods, RVQ’s progressive residual learning mechanism preserves language-shared underlying articulation patterns (e.g., consonant articulation positions, vowel formant distributions) while disentangling language-specific acoustic details (e.g., pitch modulation in tonal languages). This hierarchical codebook structure significantly enhances parameter efficiency, enabling the model to generalize to linguistically related new language variants using only limited training data.

Our results indicate that utilizing IPA-based multilingual data improves singing voice quality, while the proposed model exhibits measurable generalization potential for unseen languages. Furthermore, objective and subjective experiments confirm that Transinger significantly outperforms state-of-the-art singing synthesis methods in cross-lingual generalization. This demonstrates that multilingual aligned representations can markedly enhance model learning efficacy and robustness, even for languages not seen during training. Additionally, decomposing IPA phonemes into letters and diacritics enables the model to capture pronunciation patterns more effectively. This leads to a qualitative improvement in its generalization ability.

The remainder of this paper is organized as follows. Section 2 reviews the related work on speech synthesis and deep generative models. In Section 3, we introduce the dataset constructed in our study. The architecture of our proposed model is elaborated in Section 4. The experimental setup and results are discussed in Section 5. Lastly, Section 6 summarizes the paper and proposes future research directions.

## 2. Relate Work

### 2.1. Singing Voice Synthesis

Singing Voice Synthesis (SVS) is a specialized field of speech synthesis that generates artificial singing by modeling pronunciation rules for lyrics and analyzing pitch parameters. While sharing fundamental similarities with Text-to-Speech (TTS) systems, SVS presents unique challenges due to its musical context and expressive requirements. Historically, SVS systems evolved through two primary paradigms: concatenative synthesis using pre-recorded speech units (exemplified by Vocaloid [4]) and statistical parametric synthesis employing Hidden Markov Models (HMMs) [13,14] for phoneme transition modeling.

The advent of deep learning has revolutionized acoustic feature mapping in SVS, leading to significant advancements in synthesis quality. Pioneering work by J. Kim et al. [15] demonstrated the effectiveness of deep LSTM-RNN architectures in enhancing naturalness, particularly for Korean vocal synthesis. Subsequently, Y. Hono et al. [16] introduced a novel conditional Wasserstein GAN (cWGAN) framework, pushing the boundaries of SVS capabilities.

Recent breakthroughs in deep learning have propelled SVS to unprecedented levels of quality and expressiveness. Transformer-based architectures, particularly exemplified by HiFiSinger [17], have leveraged multi-head self-attention mechanisms to achieve superior cross-modal alignment between lyrics and musical notes, resulting in more natural and expressive synthesized singing. Concurrently, diffusion-based approaches like DiffSinger [18] have emerged as a powerful paradigm, employing stochastic differential equations to iteratively refine mel-spectrograms, thereby enhancing both audio quality and prosodic continuity. These contemporary approaches have demonstrated substantial improvements over traditional parametric methods, establishing new benchmarks in SVS performance.

### 2.2. Audio Codec

The evolution of audio coding has progressed from traditional hybrid architectures to modern neural end-to-end paradigms, significantly impacting singing voice compression capabilities. Conventional hybrid codecs like Opus [19] and Adaptive Multi-Rate (AMR) [20] combine waveform-preserving and parametric techniques through manual engineering. Opus’s dual-codec approach (SILK for speech/CELT for music) achieves balanced quality–latency tradeoffs, while AMR’s Algebraic Code-Excited Linear Prediction (ACELP) enables adaptive speech encoding. However, their reliance on handcrafted vocoder principles causes quality degradation in singing voice compression scenarios, particularly at sub-16 kbps bitrates where harmonic structures and wide-frequency vocal characteristics challenge traditional linear prediction models.

Neural audio codecs address these limitations through data-driven representation learning, particularly benefiting singing voice synthesis systems requiring high-fidelity vocal compression. By shifting acoustic feature transformation to encoder-side latent space projection, these end-to-end architectures achieve superior rate-distortion performance while maintaining computationally efficient decoding, which is a critical advantage for real-time singing voice applications. The paradigm shift from manual feature engineering to learned compression enables better preservation of singing voice nuances like vibrato, breathiness, and dynamic pitch variations that conventional codecs often discard.

Recent breakthroughs in deep learning have accelerated neural codec development through three key innovations: (1) training scale expansion from constrained speech corpora to diverse audio collections encompassing singing voices, enabling robust cross-domain generalization; (2) discrete tokenization via the Vector-Quantized Variational Autoencoder (VQ-VAE) [21], which facilitates structured latent representations crucial for encoding singing voice harmonics; and (3) integration of language model-inspired architectures using self-attention mechanisms that effectively capture long-range pitch dependencies and formant relationships in vocal signals.

State-of-the-art neural codecs demonstrate particular advantages for singing voice compression through architectural innovations. SoundStream’s [22] residual vector quantization enables multi-band fidelity preservation at ultra-low bitrates (3–6 kbps), critical for bandwidth-constrained singing voice transmission. Its structured dropout mechanism dynamically adapts to vocal complexity variations, from simple melodic phrases to operatic coloratura passages. Encodec [23] advances this through adversarial training with multi-scale spectrogram discriminators that specifically preserve singing voice timbral qualities. The integration of lightweight Transformer-based post-quantization modeling further optimizes bitrate allocation for singing voice characteristics while maintaining real-time decoding capabilities essential for interactive synthesis systems.

This technological progression establishes neural codecs as the emerging standard for singing voice compression, overcoming traditional codecs’ limitations in handling wide-band vocal expressions. The continuous-to-discrete learning paradigm enables efficient encoding of singing voice specifics—from subtle vocal fry to powerful belt registers—within low-bitrate constraints. As model architectures evolve to incorporate singing-specific feature learning and expanded vocal datasets, neural codecs are positioned to become indispensable components in next-generation singing voice synthesis pipelines, ultimately replacing conventional coding approaches through superior perceptual quality and parametric efficiency.

### 2.3. Deep Generative Models

Early generative models relied on dimensionality reduction and density estimation techniques, such as Principal Component Analysis (PCA) [24], Gaussian Mixture Model (GMM), and Independent Component Analysis (ICA) [25]. These approaches, grounded in simplified parametric assumptions, employed linear transformations or finite-component mixture models, which proved inadequate for capturing the complexity of high-dimensional data manifolds. The field evolved with the emergence of hybrid architectures that integrated generative and discriminative objectives. These included sequential state-space models (e.g., Hidden Markov Model, HMM) [26], graph-structured energy functions (e.g., Markov Random Field, MRF) [27], and bipartite stochastic neural networks (e.g., Restricted Boltzmann Machine, RBM) [28].

Variational Autoencoder (VAE) [29] introduced probabilistic latent space modeling via an encoder–decoder framework, with Kullback–Leibler (KL) divergence regularization enforcing alignment with a prior distribution. This design facilitates latent space disentanglement but often results in an excessively smooth output, sacrificing fine-grained detail and perceptual fidelity. Generative Adversarial Network (GAN) [30], by contrast, operates through an adversarial training paradigm involving a generator and discriminator. Despite their capacity for high-fidelity synthesis, GANs suffer from training instability, mode collapse, and the generation of artifact or implausible sample.

Conditional variants of VAE and GAN (for example, conditional VAE, CVAE [31]; conditional GAN, CGAN [32]) enable task-specific generation by conditioning on external inputs. To address the limitations of GAN and VAE, subsequent advancements introduced architectural and algorithmic innovations. These include Wasserstein GAN (WGAN) [33] for improved gradient stability, Boundary Equilibrium GAN (BEGAN) [34] for balancing generator–discriminator dynamics, and Normalizing VAE (NVAE) [35] for enhanced modeling of complex distributions. Hybrid frameworks such as VAE/GAN [36], Adversarial Autoencoder (AAE) [37], and CVAE-GAN have also been explored to combine the strengths of probabilistic and adversarial approaches [38].

Vector-Quantized Variational Autoencoder (VQ-VAE) [21,39] enhances traditional VAE by incorporating discrete latent variables, enabling the model to learn a finite set of embeddings. This quantization process effectively captures complex data structures, leading to improved performance in generating high-fidelity samples. By combining the probabilistic framework of VAE with discrete representation learning, VQ-VAE addresses limitations such as blurry outputs and enhances the quality of synthesized data.

Beyond VAE and GAN, diffusion-based models and flow-based architectures have emerged as powerful generative paradigms. Diffusion models progressively perturb data through a structured noise-addition process (forward diffusion) and learn to reverse this degradation via a denoising network (reverse diffusion), enabling high-fidelity sample generation [40]. Flow-based models, in contrast, employ invertible transformations to map simple base distributions (e.g., Gaussians) to complex data distributions, offering tractable likelihood estimation through bijective mappings.

These advancements have collectively elevated the capacity of generative models to synthesize high-quality, domain-specific data, with significant implications for vocal synthesis applications ranging from speech enhancement to singing voice generation.

## 3. Dataset

### 3.1. Overview

The scarcity of multilingual singing corpora featuring globally unified phonetic representations presents a fundamental bottleneck in cross-lingual singing voice synthesis (SVS) research. Current datasets like CPOP [41,42] and GTSinger [11] exhibit fragmented phoneme encoding paradigms: Chinese corpora predominantly employ Pinyin-based segmentation, while English datasets rely on ARPA phoneme standards. This linguistic compartmentalization creates systemic incompatibilities in multilingual modeling, particularly hindering cross-lingual phoneme composition and style transfer capabilities.

Our language selection and dataset construction follow rigorous scientific principles rooted in the geographical distribution of singing voice synthesis technologies. The Asian market dominates SVS adoption due to its unique digital content consumption culture and advanced receptiveness to music technologies. We strategically selected Chinese, Japanese, and Korean as primary linguistic representatives of East Asia, where these languages exhibit fundamentally distinct prosodic systems: Chinese employs tonal phonology, Japanese utilizes mora-timed rhythm, and Korean features complex consonant clusters. This intentional diversity not only enables models to learn cross-lingual articulation patterns but also enhances their robustness against phonological variations.

The selected languages exhibit substantial phonological contrasts, each imposing unique challenges on singing voice modeling. Chinese is a tonal language in which lexical meaning is directly tied to pitch contour, requiring precise alignment between tonal trajectories and musical melodies. Japanese features a mora-timed rhythmic structure, where each mora serves as a timing unit, demanding highly regular phoneme-to-beat synchronization. Korean introduces complex consonant clusters and a rich inventory of stop consonants, which complicates the modeling of articulatory clarity and spectral transitions. English, in contrast, is stress-timed and characterized by frequent consonant onsets and syllabic stress alternations, requiring the model to capture dynamic prosodic variation and complex phonotactics. These differences result in diverse acoustic manifestations across languages, significantly complicating efforts to build a unified SVS model.

Our dataset also reflects substantial phonetic variation at the segmental level: retroflexes and tone markers dominate Chinese samples, nasalized vowels are prevalent in Korean recordings, and glides and diphthongs are frequently observed in English utterances. This distribution not only highlights the inherent diversity in cross-linguistic articulatory patterns but also enriches the model’s phonetic representation space, thereby providing a broader foundation for multilingual generalization and phoneme recombination.

Our linguistic analysis of East Asian pop music revealed pervasive code-switching phenomena, particularly frequent English phrase insertions within native-language lyrics (e.g., Korean choruses with English hooks). An analysis of lyrics from top-ranked Korean girl group tracks on national digital charts shows that English content accounts for more than 40% of total phrase-level segments [43]. This real-world hybridity necessitates modeling linguistic transitions between Asian languages and English, motivating our inclusion of English data. Such mixed-language scenarios create unique technical challenges in maintaining prosodic consistency across language boundaries within single musical phrases.

Audio files were sliced into 5–20 s clips based on phonetic boundaries, to ensure phoneme alignment accuracy. Cross-validation was conducted between extracted F0 contours, IPA transcripts, and original musical scores. Multi-expert reviewed process for linguistic accuracy and singing technique validation.

### 3.2. Symbol

In phonetic transcription, segments are generally represented by one or more symbols from the International Phonetic Alphabet (IPA). These symbols are primarily divided into two categories: letters and diacritics. For instance, in English, [c^w^] consists of the letter [c] and the labialization diacritic [^w^].

The decision to separate IPA letters and diacritics is grounded in fundamental phonological principles. In phonology, a complex phoneme is typically viewed as a composition of a base sound and one or more phonetic modifiers. IPA letters usually encode the core articulatory gesture of a sound—determining the place of articulation (e.g., bilabial, alveolar, velar) and manner of articulation (e.g., plosive, nasal, fricative). For instance, [p], [t], and [k] are base plosives. In contrast, diacritics denote secondary articulatory features such as aspiration [^h^], labialization [^w^], or palatalization [^j^]. These modifiers are combinatorially reusable across base phones. For example, aspiration [^h^] can be applied to various plosives to form [p^h^], [t^h^], and [k^h^]. If the model were to treat [p^h^] and [t^h^] as independent, atomic units, it would struggle to generalize to unseen combinations like [k^h^]. Our decomposition strategy allows the model to separately learn the acoustic patterns of base letters and diacritics. When encountering unseen phonemes during inference (e.g., [k^h^]), the model can synthesize them by recombining previously learned components—supporting zero-shot compositional generalization. This mechanism directly addresses the challenges of cross-lingual data sparsity and phoneme diversity.

Regarding letters, in addition to the 107 letters recognized in the latest version of the International Phonetic Alphabet (IPA), we have also introduced SVS-specific symbols, such as those for silence (SP) and aspiration (AP). It is important to note that certain phonemes appear in combined forms and are pronounced as a single unit. For diphthongs like [aw], there is a smooth and continuous transition between the sounds [a] and [w] during pronunciation, without a distinct boundary. Therefore, in our system, we treat such diphthongs as a single phoneme and assign a dedicated symbol to them, ensuring that this natural and coherent transition is accurately captured during speech synthesis.

Regarding diacritics, we have refined the official IPA set of 40 diacritics. Based on experimental observations, tones and word accents have minimal impact on the final singing voice. Therefore, we have omitted these related diacritics from our SVS system. This simplification not only enhances system efficiency but also helps maintain the naturalness of the synthesized singing voice. Our experiments demonstrate that this optimized IPA system effectively captures key phonetic features across different languages, providing a robust foundation for phonemic representation in multilingual singing voice synthesis. Furthermore, this enhancement enables better adaptability and scalability when handling cross-lingual singing voice synthesis.

**Figure 1:**
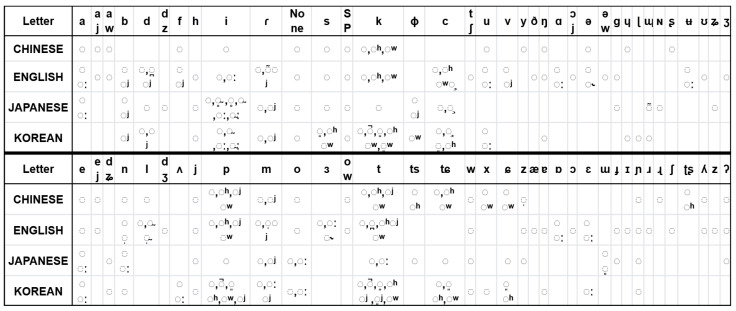
Cross-linguistic distribution of IPA letters and diacritic combinations across the four languages in the dataset (Chinese, English, Japanese, and Korean). Note: A dotted circle represents a base letter with no diacritic. Any elements not covered in the dataset are omitted from the table.

As illustrated in Figure 1, the cross-linguistic statistical distribution of IPA components reveals significant overlaps in both letters and diacritics, which are often shared across languages but appear in different combinations. These overlaps form a critical basis for two key capabilities: (1) cross-linguistic generalization through the parameterized recombination of phonetic primitives, and (2) zero-shot inference on unseen languages via the systematic composition of learned phonological features.

### 3.3. Alignment

Based on the audio files of four languages (Chinese, English, Japanese, and Korean) selected from the GTSinger dataset, our preprocessing pipeline involved the following phases:

The lyrics were first processed through GTSinger’s specialized phonetization system, which systematically converts text into contextualized phoneme sequences. This framework integrates domain-specific dictionaries for precise articulation mapping and predefined silent markers for breathing patterns and musical phrase segmentation. This process preserved the dataset’s original phoneme-level technique annotations for six vocal styles (mixed voice, falsetto, breathy voice, etc.).

**Figure 2:**
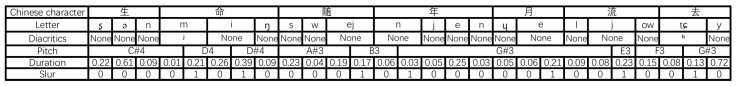
Illustration of a labeled sample in the multilingual singing voice synthesis dataset. The “Chinese character” row displays the lyrics corresponding to the phonemes in the “Letter” row.

Then, we used the Montreal Forced Aligner (MFA) [44], a widely used tool for automatic phoneme-to-audio alignment, with music-adapted acoustic models to initialize temporal boundaries and map phonemes to audio. Then, we integrated F0 contours and MIDI scores for frame level and pitch synchronous alignment, and solved rhythm irregularities via beat tracking of original accompaniments. Three certified music annotators cross-validated the alignments in Praat (V6.4.31), a software for phonetic analysis, by adjusting boundaries of vibrato-affected phonemes, segmenting silent regions (0.3 s threshold for breath detection), and conducting cross-language validation. This ensured alignment accuracy and reliability across languages, providing high-quality annotated data for music speech analysis and processing. As illustrated in Figure 2, an example from the dataset is presented.

## 4. Transinger

**Figure 3:**
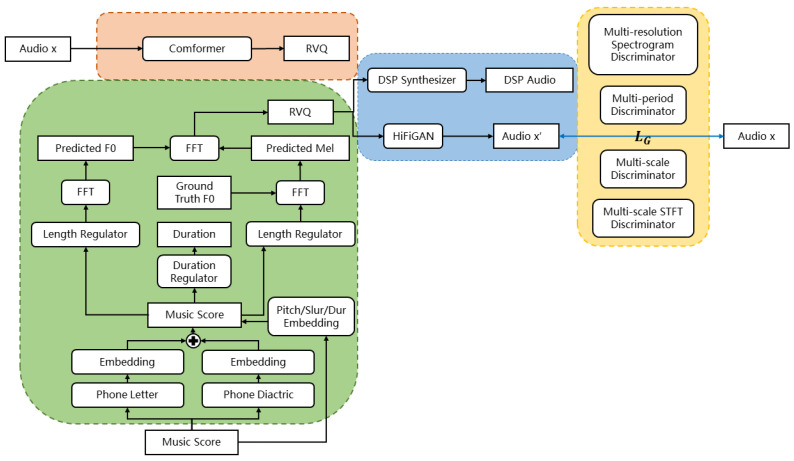
Overall model architecture of Transinger. The model consists of four components: (1) the posterior encoder (orange), which processes the input audio to produce posterior quantized representations via a conformer and residual vector quantization (RVQ); (2) the prior encoder (green), which generates prior quantized representations from the music score and predicted pitch using a sequence of phoneme embedding, duration modeling, and acoustic prediction modules; (3) the generative network G (blue), which synthesizes waveforms from the predicted acoustic features; and (4) the discriminative network D (yellow), which evaluates the generated audio using multiple domain-specific discriminators to guide adversarial training.

The overall model architecture of Transinger is presented in Figure 3. It adopts the CVAE structure from VISinger2 [1], which includes a posterior encoder, a prior encoder, and a decoder. In the traditional CVAE setup, $z^{\prime }$ and *z* are sampled separately from the prior and posterior distributions, with constraints imposed to minimize the discrepancy between them. Then, the final latent representation *z* is drawn from the posterior distribution and input into the *G* network to produce audio. We have enhanced the encoding component by introducing RVQ for encoding, which allows for a more fine-grained and flexible discrete representation, thereby improving the generation quality.

### 4.1. Posterior Encoder

The original model uses multiple convolutional layers to extract audio features. However, its flexibility and expressiveness are insufficient for diverse background requirements, and its ability to learn temporal dependencies is suboptimal. The model’s adaptability in different contexts is poor, making it inadequate for handling complex background conditions.

Inspired by MuCodec [45], our posterior encoder is primarily composed of several conformer blocks and Residual Vector Quantization (RVQ). Transformers excel at capturing global interactions, while CNNs are highly effective in extracting local features. The Conformer combines these strengths to model both local and global dependencies in audio sequences with high parameter efficiency. Since audio signals inherently contain complex temporal and spectral information, the Conformer’s hybrid architecture enables it to simultaneously capture fine-grained local details and overarching global patterns, making it highly efficient for tasks such as audio generation, synthesis, and recognition.

In our model, Residual Vector Quantization (RVQ) is employed to efficiently encode and compress audio features. RVQ learns a discrete codebook and maps continuous audio features to the most appropriate quantization vectors, enabling more fine-grained latent representation for the generation module. This approach not only enhances the model’s efficiency in representing and generating audio but also yields a more compact encoding that facilitates efficient storage and transmission. In line with the requirements of our model standardization task, employing RVQ enables a more fine-grained and flexible discrete representation while preserving key audio details, thereby providing high-quality latent representations for the subsequent generation module.

### 4.2. Prior Encoder

To effectively capture phonetic characteristics, the text encoding module in the prior encoder embeds IPA letters and diacritics separately and combines them through element-wise addition. This method ensures flexible phoneme encoding while preserving distinct phonetic features, enabling the model to learn separate feature representations for each component. Consequently, the model enhances phoneme encoding accuracy and flexibility and is better equipped to recognize and generate phonemes not encountered during training.

Furthermore, this decomposition strategy significantly improves modeling efficiency and generalization. Treating each IPA phoneme as an indivisible symbol would lead to a large and sparse label space due to the vast number of possible combinations across languages. By factorizing the phoneme representation into letter and diacritic components, the model operates over a more compact embedding space with enhanced parameter sharing. Articulatory features such as aspiration or palatalization—encoded via diacritics—can be shared across multiple base letters, allowing the model to generalize more effectively to new phonetic compositions.

This structure also supports compositional zero-shot generalization. During inference, the model may encounter phonemes that were unseen during training as complete units, but whose constituent parts were observed separately. For instance, the phoneme [d̪] may not appear in the training corpus, yet both [d] and the dentalization diacritic [ ̪] might be individually learned. By recombining the learned embeddings of these components, the model can synthesize novel phonemes in a principled and interpretable way.

In the original VISinger2 [1] model, the prior encoder processes discrete music scores, while the posterior encoder handles continuous audio signals. The asymmetry between the discretely quantized prior (mapped to a continuous space) and the posterior (modeled as a continuous Gaussian distribution) can cause several issues. The posterior distribution may fail to align with the quantized prior space, optimizing the Kullback–Leibler divergence becomes more challenging, and the latent space structure may become inconsistent. To resolve these issues, we have enhanced the original model by adding a Residual Vector Quantization (RVQ) module at the end of the prior encoder. This modification improves the consistency between the prior and posterior latent representations, thereby boosting the model’s overall generation performance.

### 4.3. Prior–Posterior Alignment

In VISinger2 [1], the model calculates the Kullback–Leibler (KL) divergence to measure the difference between the prior distribution $z^{\prime }$ and the posterior distribution *z*, showing how close the posterior is to the prior. The prior distribution is characterized by the mean and standard deviation of the latent variable *z*, whereas the posterior distribution is defined by the mean and standard deviation of the audio feature representation generated by the encoder. Acting as a regularization term, the KL divergence restricts the posterior–prior distribution discrepancy, ensuring the posterior does not deviate too much from the prior.

We depart from the conventional use of KL divergence in the CVAE architecture and instead propose a composite loss function with three distinct components. This customized loss function not only ensures meticulous and robust alignment between the prior quantization $q_{\mathrm{prior}}$ (derived from the music score, including the phoneme sequence) and the posterior quantization $q_{\mathrm{posterior}}$ (derived from the ground-truth acoustic features) in the latent space, but more importantly, it reinforces the consistency of phoneme-level information across these two representations, thereby improving the accuracy of phoneme alignment.

(1) $$L=\lambda_{1}L_{\mathrm{MSE}}+\lambda_{2}L_{\cos}+\lambda_{3}L_{L1}$$

1. Mean Squared Error (MSE) captures the Euclidean distance between the prior and posterior quantizations. By penalizing large deviations, it ensures coarse-grained structural consistency in the latent space—effectively aligning the overall acoustic characteristics (e.g., duration, energy envelope, spectral balance) between the music score and the actual audio. This forms a foundational match before finer distinctions are addressed.
  (2) $$L_{\mathrm{MSE}}=\frac{1}{N}\sum_{i=1}^{N}{∥q_{\mathrm{prior},i}-q_{\mathrm{posterior},i}∥}^{2}$$
2. Cosine Similarity measures the angular difference between latent vectors, thereby promoting alignment in vector direction. In the context of phonetic alignment, this encourages consistency in semantic identity—particularly important for capturing phoneme categories (e.g., vowels vs. consonants) and for supporting IPA-based decomposition strategies, where both symbol and modifier components must align directionally for accurate sub-phoneme modeling.
  (3) $$L_{\cos}=1-\frac{\sum_{i=1}^{N}q_{\mathrm{prior},i}\cdot q_{\mathrm{posterior},i}}{∥q_{\mathrm{prior},i}∥∥q_{\mathrm{posterior},i}∥}$$
3. L1 loss computes the absolute element-wise differences between the prior and posterior quantizations. Compared to MSE, L1 loss is less sensitive to outliers and extreme deviations. In our singing voice synthesis setting, this loss help stabilize the alignment of phonetic attributes across latent dimensions, particularly under cross-lingual variation and expressive singing conditions. Its element-wise formulation supports more consistent matching of localized phonetic traits (e.g., voicing or articulation), without being dominated by occasional large errors. While not explicitly enforcing phoneme correctness, it can contribute to a more stable learning process in challenging alignment scenarios.
  (4) $$L_{L1}=\frac{1}{N}\sum_{i=1}^{N}\left|q_{\mathrm{prior},i}-q_{\mathrm{posterior},i}\right|$$

We combine these three loss terms with appropriate weights, $\lambda_{1}$, $\lambda_{2}$, and $\lambda_{3}$, for distance, directional consistency, and robustness. This fine-grained alignment strategy serves as a foundation for high-quality phoneme modeling and is particularly crucial for cross-lingual singing voice synthesis tasks that require precise learning of phoneme pronunciation rules and generalization to unseen languages.

### 4.4. Generator Network

The generative network *G* samples from the learned conditional distribution P(x|z) to reconstruct audio $x^{\prime }$. Its decoder, matching VISinger2’s architecture, mainly comprises a DSP Synthesizer and HiFiGAN modules [46]. The decoder excels at generating high-fidelity, natural audio by effectively capturing temporal dependencies and complex features.

### 4.5. Discriminator Network

The discriminator distinguishes real training data from synthetic data. In the original VISinger2 [1] framework, discriminators were based on UnviNet’s Multi-Resolution Spectrogram Discriminator (MRSD) [47] and HiFi-GAN’s Multi-Period Discriminator (MPD) and Multi-Scale Discriminator (MSD) [46]. We have added Encodec’s MS-STFTD [23], which processes audio signals at multiple scales. The MS-STFTD uses a multi-scale complex STFT representation to capture diverse time–frequency features in audio signals, enhancing the model’s performance across scales.

The MS-STFTD improves adversarial training, boosting the quality of generated audio, particularly for complex audio events and variations. It calculates STFT features at multiple frequency resolutions, capturing the audio signal’s global structure and local details. This ensures the generator meets overall audio quality standards and accurately reproduces subtle audio variations. Moreover, by combining the perceptual loss from multi-scale discrimination, this module enhances the generation model’s ability to capture intricate time–frequency features, increasing the realism and nuance of the generated audio. Along with the existing adversarial loss and relative feature matching loss, this cooperative effect makes the training process more stable and efficient, ultimately improving the overall quality of the generated audio.

## 5. Experiments and Results

### 5.1. Cross-Language Generalization

To evaluate Transinger’s effectiveness and assess how multilingual training affects synthesis quality, we compared the following systems:

- Model 1: Transinger, our proposed multilingual singing voice synthesis model.
- Model 2: VISinger2 [1], a variant of VISinger2, where the prior encoder was adapted to support IPA-based phoneme encoding.

We conducted experiments using two datasets:

- Corpus 1 for monolingual training: This dataset contains only Chinese-language singing recordings, totaling 15.7 h at a 48 kHz sampling rate.
- Corpus 2 for multilingual training: This dataset includes all languages in our dataset, totaling 40.9 h at a 48 kHz sampling rate.

This study adopts a comprehensive evaluation framework that integrates both subjective and objective assessments to validate our self-constructed multilingual singing synthesis dataset. In our experimental design, evaluation samples were selected from the test set using stratified random sampling based on language proportions. This method ensures that the sample distribution accurately reflects the linguistic composition of the original dataset.

Our evaluation approach is based on a dual-dimensional validation method:

Subjective Evaluation: We assembled a panel of 15 native speakers representing four language groups (Chinese, English, Japanese, and Korean) to assess the samples using the internationally recognized Mean Opinion Score (MOS) system. Each evaluator independently rated the samples on two dimensions: pronunciation and expressiveness MOS. The expressiveness MOS was designed to take into account aspects such as naturalness, sound quality (e.g., clarity and dynamic range), and expressive characteristics (e.g., rhythm, vibrato extent/rate, articulation, emotional richness, and timbre fidelity), providing a more comprehensive evaluation of the synthesized speech. Ratings were provided on a 1 to 5 scale with a minimum increment of 0.5, where 5 indicates the highest quality. To ensure objectivity, the evaluation process was conducted using a double-blind design. In addition to MOS scoring, we conducted an ABX preference test to further capture perceptual differences between models. In this test, listeners were presented with a pair of audio samples (one from each model) and asked to choose which one sounded better. The three possible responses were as follows: (1) 1st is better; (2) 2nd is better; (3) neutral

Objective Evaluation: The synthesized singing voices were quantitatively assessed through three metrics: Word Error Rate (WER), F0 Root Mean Square Error (F0_RMSE), and Voiced/Silence Error Rate (VS_E).

- Word Error Rate (WER): Using Whisper [48] to transcribe the synthesized audio, we compute the Word Error Rate (WER) by comparing the transcription with the reference text. This metric reflects the pronunciation accuracy and intelligibility of the singing voice. Additionally, words that are written differently but share the same pronunciation are also considered correct.
- F0 Root Mean Square Error (F0_RMSE): This metric quantifies the deviation between the predicted and reference fundamental frequency (F0) values, providing insight into the pitch accuracy and overall prosody of the synthesis.
- Mel Frequency Cepstral Distortion (MCD): MCD is a metric used to evaluate the quality of synthesized speech by quantifying the difference between the mel cepstral coefficients of the synthesized speech and a reference. It measures the spectral similarity between two speech signals, indicating how well the synthesized speech matches the spectral characteristics of the reference.
- Voiced/Silence Error Rate (VS_E): This measures the error rate in determining whether a frame is voiced (i.e., contains vocalization) or silent. It is critical for evaluating the temporal consistency and proper activation of the singing voice.
- Emotional Similarity (ESIM): Emotion2vec [49] as an evaluation metric for emotional similarity. This approach first encodes speech signals into emotion embeddings, then computes the cosine similarity between the embeddings of the reference and generated speech. A higher similarity indicates better alignment in emotional expression.
- Dynamic Range (DR): DR refers to the difference in loudness between the quietest and the loudest parts of an audio signal. A higher dynamic range indicates greater expressiveness and more pronounced amplitude variation.

This comprehensive evaluation methodology allows for an in-depth analysis of the synthesized singing voices, encompassing both human perceptual and computational aspects.

As evidenced by Table 1, Model 1 (Transinger) outperforms Model 2 (modified VISinger2 [1]) in most evaluated metrics. In the monolingual Chinese test (Corpus 1), Model 1 shows a slightly lower word error rate (WER) than Model 2 (35.64% vs. 38.61%), though the difference is not statistically significant. However, for pronunciation quality as measured by the Mean Opinion Score (MOS), Model 1 scores significantly higher at 3.61 (±0.07) compared to Model 2’s 3.46 (±0.08), with a *p*-value of less than 0.01. This indicates that while the overall word accuracy was comparable, Model 1’s pronunciation was perceived as more accurate and natural even in the monolingual setting. The advantages of Model 1’s architecture become even more pronounced in the multilingual condition (Corpus 2). Here, it achieves a Chinese WER of 22.77%—a 14.81% relative error reduction compared to Model 2’s 26.73% (*p* < 0.05). This superiority is also reflected in the Pronunciation Mean Opinion Score (MOS), where Model 1 again outperformed Model 2 (3.69 vs. 3.59, *p* < 0.05), confirming the effectiveness of its IPA-based representation for enhancing cross-lingual generalization. The consistency between the objective WER results and the subjective MOS ratings provides strong evidence of the model’s superior capability in producing intelligible and accurate pronunciations. Model 1 trained on the multilingual corpus (Corpus 2) achieves a WER of 22.77% (±2.11) in the Chinese test set, compared to 35.64% (±2.37) for the same model trained solely on the monolingual Chinese corpus (Corpus 1). This represents a relative error reduction of approximately 36% (*p* < 0.001). The significant improvement suggests that exposure to phonetic and prosodic patterns from multiple languages enhances the model’s ability to generalize pronunciation rules.

For Corpus 2, Model 1 achieves an F0_RMSE of 5.31, representing a 22.8% improvement over Model 2’s 6.88. This indicates more precise pitch control. The improvement in F0_RMSE can be attributed to the Conformer’s ability to jointly model local rhythmic patterns and global pitch contours, as well as the robustness of RVQ in capturing continuous pitch variations. However, Transinger shows only a slight advantage in MCD (6.593 vs. 6.700), indicating that there is still room for improvement in spectral reconstruction quality. The VS_E rises from 5.56% in the monolingual setting to 9.75% under multilingual training. While the increased syllabic diversity enhances the model’s generalization to rhythm boundaries across languages, it also introduces timing mismatches in certain cases. Designing guidance mechanisms to improve fine-grained rhythmic alignment remains an important direction for future work.

In the Chinese dataset, Transinger achieves an ESIM of 3.76, higher than Model 2’s 3.15, likely due to better learning of fine-grained details such as nasalization, vibrato, and vowel transitions. In contrast, its slightly lower dynamic range (DR) may result from loudness normalization across languages, which could reduce language-specific dynamic expressiveness. Transinger also achieves an expressiveness MOS of 3.37 ± 0.06 (Chinese) and 3.33 ± 0.09 (overall), about 0.3 points higher than Model 2 (*p* < 0.001). This suggests that cross-lingual generalization was achieved without sacrificing within-language expressiveness. This can be attributed to the RVQ module’s ability to retain essential features—such as timbre, formants, and rhythm—while compressing shared patterns across languages, effectively capturing cross-lingual expressive cues like vibrato, breathing, and glide.

**Figure 4:**
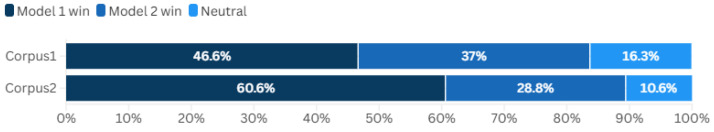
ABX test results comparing Model 1 (Transinger) and Model 2 (VISinger2 variant).

As shown in Figure 4, our model (Model 1) is consistently preferred over the baseline (Model 2), with a higher win rate in both the monolingual (46.6%) and multilingual (60.6%) settings. It is worth noting that the performance on non-Chinese languages tends to be slightly lower across most metrics. Striking the right balance between shared representations (to promote efficiency and cross-lingual transfer) and language-specific components (to ensure accuracy) remains a key challenge.

### 5.2. Zero-Shot Language Generalization

To validate the effectiveness of the proposed multilingual phoneme encoding strategy in generalizing to unseen languages, we designed two experimental setups:

1. IPA-Merged Encoding: Based on the International Phonetic Alphabet (IPA), this method combines letters and diacritics into a single encoding unit, treating each phoneme as an indivisible entity.
2. IPA-Disentangled Encoding: This approach separately embeds IPA letters and diacritics, followed by feature fusion.

To assess cross-linguistic accuracy, we trained models using Chinese, Japanese, and Korean data from our dataset and selected English from the same dataset as the test language. A total of 32 test segments were randomly chosen, encompassing the following scenarios:

1. Phoneme combinations not present in the training set, where the constituent letters and diacritics exist in different languages within the training samples.
2. Phoneme combinations absent from the training set, with one constituent (either letter or diacritic) present in the dataset and the other absent.
3. Phoneme combinations entirely unseen by the model, including diacritics never encountered during training.

Since the text encoder cannot process phonemes unseen during training, we incorporated these phonemes into the embedding vectors prior to training to address the aforementioned scenarios.

Objective Evaluation: We segmented the synthesized speech into words or phrases and used the Whisper model [48] to detect each word, calculating the Phone Error Rate (PER). In this calculation, detections that match the correct word or its homophones were not counted as errors. Moreover, if only a subset of the phonemes in a word is correctly detected, those correctly identified phonemes are also considered accurate. We employed the following metrics to assess speech quality:

- Fundamental Frequency Root Mean Square Error (F0-RMSE): Measures the difference between estimated and true fundamental frequency values.
- Mel Spectral Distortion (MSD): Assesses the degree of difference between Mel spectrograms.
- Voiced\Silence Error Rate (VS_E): Calculates the proportion of errors in classifying voiced and silence segments.
- Speaker Similarity Assessment (SA): Quantifies the similarity between synthesized speech and the target speaker.

Subjectively, a Phoneme Recognition Rate (PRR) evaluation was conducted using a panel of eight native Chinese speakers, all of whom were proficient in English. Participants were instructed to listen to the synthesized audio samples and transcribe the phonemic content they perceived. Transcriptions that corresponded to the reference phonemic sequences—or that conformed to acceptable phonetic variants according to established criteria—were considered accurate. This rigorous assessment provides a quantitative measure of the system’s phoneme recognition capabilities.

The experimental results validate the effectiveness of our proposed multilingual speech synthesis (SVS) strategy (in Table 2). Compared to language-specific phoneme representations, using International Phonetic Alphabet (IPA) phonemes significantly enhances cross-language synthesis performance. Moreover, breaking down IPA phonemes into letters and diacritics further improves cross-language performance. These findings highlight the substantial application value of separating IPA phonemes in multilingual speech synthesis.

## 6. Conclusions

In this study, we introduce Transinger, a novel cross-lingual singing voice synthesis framework that enables the synthesis of singing voices in languages not encountered during training by better learning latent features for enhanced language generalization. We establish IPA-phoneme-based pronunciation rules tailored for Singing Voice Synthesis (SVS) tasks, and construct a multilingual singing dataset through systematic integration of open-source resources coupled with precise segmentation and calibration. This dataset features cross-linguistically consistent phoneme-level annotations, providing enhanced robustness assurance for model training through its phonetically unified representation across languages. We have pioneered a method that decomposes IPA phonemes into letters and diacritics, enabling refined feature extraction and achieving a qualitative leap in performance for inference on unseen languages.

Our dataset covers Indo-European languages, Sino-Tibetan languages, and the Altaic–Austronesian mixed language family, encompassing the vast majority of the global population. By conducting experiments across such diverse linguistic backgrounds, we have validated the generalizability of our approach. Whether dealing with mainstream languages or relatively less common ones, the method demonstrates excellent cross-lingual transfer capabilities and phonetic consistency, fully showcasing its broad applicability and robustness in cross-lingual singing voice synthesis tasks.

Our experimental results demonstrate that Transinger not only facilitates effective cross-lingual capabilities but also maintains strong performance in languages already present in the dataset, outperforming baseline models. Furthermore, ablation studies have validated the efficacy of our proposed method.

In future work, we plan to expand our dataset by incorporating additional languages and increasing the overall data volume to further enhance performance. We also aim to explore more advanced zero-shot language generalization techniques to improve the quality of synthesized singing voices. Moreover, by decomposing IPA phonemes into letters, and diacritics, we can better capture the intricate characteristics of pronunciation. Although multilingual training enhances expressive capabilities across languages, it may slightly compromise the accuracy of certain audio aspects. Addressing this trade-off will be a key focus of our future research.

## Figures and Tables

**Table 1:** Experimental results in terms of subjective mean opinion score (MOS) and objective metrics.

Data Scope	Model	Language	WER ↓	Pronunciation ↑	F0_RMSE ↓	MCD ↓	VS_E ↓	ESIM ↑	DR ↑	Expressiveness ↑
Corpus 2	Model 1	CN	22.77 (±2.11)%	3.69 (± 0.05)	5.01	6.593	9.75%	3.76	77.47	3.37 (±0.06)
		ALL	23.98 (±1.94)%	3.43 (±0.10)	5.31	7.002	9.68%	3.44	78.87	3.33 (±0.09)
	Model 2	CN	26.73 (±2.21)% *	3.59 (± 0.06) *	6.70	6.700	10.43%	3.15	80.86	3.08 (±0.07) ***
		ALL	28.11 (±2.03)% **	3.41 (±0.11) ns	6.88	7.016	10.52%	2.67	81.69	3.07 (±0.09) ***
Corpus 1	Model 1	CN	35.64 (±2.37)% ***	3.61 (± 0.07) *	4.454	6.526	5.56%	3.36	78.62	3.26 (±0.09) *
	Model 2	CN	38.61 (±2.40)% ns	3.46(± 0.08) ^‡^	4.812	6.651	5.92%	3.22	80.87	3.02 (±0.11) ^‡^
Ground Truth		CN	7.61 (±1.40)% ***	4.77 (± 0.04) ***	/	/	/	/	82.66	4.68(± 0.08) ***
		ALL	8.35 (±1.31)% ***	4.76 (± 0.05) ***	/	/	/	/	83.48	4.71 (± 0.07) ***

The results for WER and MOS are reported with 95% confidence intervals (CI). WER confidence intervals are computed using the Wilson score interval method, whereas MOS confidence intervals are derived through bootstrapping. Statistical significance is reported for two distinct baseline comparisons. First, an asterisk (*) indicates a significant difference when a result is compared to our proposed multilingual model (Model 1 under Corpus 2 for the respective language), with the following levels: * *p* < 0.05, ** *p* < 0.01, *** *p* < 0.001. Second, a dagger indicates a significant difference when a result is compared to our proposed monolingual model (Model 1 under Corpus 1), with the following levels: ^‡^ *p* < 0.01. Non-significant (*p*≥ 0.05) results are marked as ns. For each metric, ↑ indicates that a higher value is better, while ↓ indicates that a lower value is better.

**Table 2:** Cross-language performance assessment of phoneme encoding approaches.

Data Format	PER	PRR	F0-RMSE	MSD	VS_E	SA
Merged L&D	1.127%	3.105%	7.4398	75.1204	16.3%	9.01%
Split (L + D)	9.219%	22.65%	6.4204	59.2089	12.86%	15.89%

## Data Availability

The datasets presented in this article are not readily available because the data are part of an ongoing study. Requests to access the datasets should be directed to the corresponding author, 202221147064@njtech.edu.cn.
